# Moving Food Assistance into the Digital Age: A Scoping Review

**DOI:** 10.3390/ijerph19031328

**Published:** 2022-01-25

**Authors:** Nina M. Martin, Daniel J. Barnett, Lisa Poirier, Samantha M. Sundermeir, Melissa M. Reznar, Joel Gittelsohn

**Affiliations:** 1 Human Nutrition Program, Department of International Health, Johns Hopkins Bloomberg School of Public Health, Baltimore, MD 21205, USA; lpoirie4@jhu.edu (L.P.); srex2@jh.edu (S.M.S.); jgittel1@jh.edu (J.G.); 2 Department of Environmental Health and Engineering, Johns Hopkins Bloomberg School of Public Health, Baltimore, MD 21205, USA; dbarnet4@jhu.edu; 3 Department of Interdisciplinary Health Sciences, Oakland University School of Health Sciences, Rochester, MI 48309, USA; reznar@oakland.edu

**Keywords:** food security, food assistance, digital applications, digital technology, digital health

## Abstract

One of the most basic needs globally, food assistance refers to the multitude of programs, both governmental and non-governmental, to improve food access and consumption by food-insecure individuals and families. Despite the importance of digital and mobile Health (mHealth) strategies in food insecurity contexts, little is known about their specific use in food assistance programs. Therefore, the purpose of this study was to address that gap by conducting a scoping review of the literature. Keywords were defined within the concepts of food assistance and digital technology. The search included relevant peer-reviewed and grey literature from 2011 to 2021. Excluded articles related to agriculture and non-digital strategies. PRISMA guidelines were followed to perform a partnered, two-round scoping literature review. The final synthesis included 39 studies of which most (84.6%) were from the last five years and United States-based (93.2%). The top three types of articles or studies included text and opinion, qualitative research, and website, application, or model development (17.9%). The top three types of digital tools were websites (56.4%), smartphone applications (20.5%), and chatbots (5.1%). Nineteen digital features were identified as desirable. Most tools included just one or two features. The most popular feature to include was online shopping (*n* = 14), followed by inventory management, and client tracking. Digital tools for individual food assistance represent an opportunity for equitable and stable access to programs that can enhance or replace in-person services. While this review identified 39 tools, all are in early development and/or implementation stages. Review findings highlight an overall lack of these tools, an absence of user-centered design in their development, and a critical need for research on their effectiveness globally. Further analysis and testing of current digital tool usage and interventions examining the health and food security impacts of such tools should be explored in future studies, including in the context of pandemics, where digital tools allow for help from a distance.

## 1. Introduction

Mobile Health (mHealth) and digital application technology have revolutionized public health [1,2,3,4]. Digital interventions have been shown to successfully improve health behaviors, ranging from smoking cessation to diet [5,6] and health outcomes, including weight loss [7,8,9,10]. A strength of digital strategies lies in the near ubiquity of social media and digital communication platforms, which allows for the cost-effective and sustainable engagement of peers for driving individual- and group-level behavior change [2,11,12,13,14]. Digital ‘networking’ in the context of mHealth relates to building social networks to improve relationships between patients and healthcare providers, which includes role modelling, perceived self-efficacy, personalized feedback, and health education [15]. Digital networking to reinforce or drive behavior has been tested on a wide range of strategies from diabetes management in adolescents [16], to weight reduction [17] and diabetes prevention [18], to increasing consumer spending through group discounts [19].

Food assistance and distribution remains one of the most basic needs globally, both in disaster and non-disaster settings. Food assistance refers to the multitude of programs, both governmental and non-governmental, to improve access and consumption of food by food-insecure individuals and families [20,21]. In the United States, this includes national programs such as the Supplementary Nutritional Assistance Program (SNAP) and the Special Supplemental Nutrition Program for Women, Infants, and Children (WIC) [21,22]. SNAP and WIC beneficiaries receive stipends to buy foods at participating outlets, such as grocery stores and farmers’ markets. SNAP- and WIC-eligible participants must apply and meet specified qualifications to receive these food security benefits. At the state and regional level, food banks are responsible for the large-scale distribution of food to local food pantries. Food pantries oversee the distribution of food and sometimes education to food-insecure clients in their cities and neighborhoods.

Digital strategies for food assistance program operations could improve and maintain services in and outside of disaster settings. During the COVID-19 pandemic, however, food assistance programs stalled or shut down because of the lack of existing resources to switch from in-person or paper-based systems to digital platforms, while there has been a surge in food insecurity [23,24,25,26,27]. For example, many food banks experienced supply shortages, and many were forced to close in the face of their lack of resources to build digital platforms in a time-sensitive fashion [26,27]. Other fields have applied insights from COVID-19 to mature their digital technologies; however, these efforts have been notably lacking for food assistance programs, despite the potential benefits that could be achieved at a relatively low cost [28,29,30,31,32,33]. Solutions that have been applied elsewhere that could benefit food assistance programs are, for example, platforms for SNAP users to purchase food online from free-to-discounted sources of food or improving communications between food pantries and their network of volunteers, clients, and stakeholders. Some reasons for this lag are outdated views that low-income residents do not have access to digital technology and use of antiquated technology in use prior to COVID-19. These perceptions are amplified in turn at the pantry level, where there is little time and resources available, and operations are mostly run by volunteers.

There is little information on digital strategies currently in use for food assistance programs. To define the current state of digital strategies for food assistance, this study aims to: (1) define the scope and type of research regarding digital tools used for food assistance; (2) describe the digital tools in terms of key functions, platform, intended users, and beneficiaries; and (3) identify key gaps in research and development and make recommendations accordingly for future work.

## 2. Materials and Methods

To explore the current literature surrounding digital interventions for addressing food assistance, we conducted a scoping review of the peer-reviewed and grey literature in August–October 2021. We searched PubMed, Embase, Cinahl, Scopus, and Web of Science to identify studies published from 2011 to 5 October 2021. Search terms were developed using a combination of controlled vocabulary and keywords to define the concepts of food assistance and digital technology, and terms were adapted for use in each database. Similar search terms were used to search the grey literature in Google, Science.gov, Worldwidescience.org, the World Food Programme, United States Agency for International Development, UNICEF, and Feeding America websites. A summary of search terms and results are presented in Table 1.

Using the PRISMA (Preferred Reporting Items for Systematic Reviews and Meta-Analyses) Checklist [34] as a guide throughout, all citations were imported into the EndNote citation management system (Clarivate Analytics, Philadelphia, PA, USA) to remove duplicate records, and then imported into Covidence systematic review software (Veritas Health Innovation, Melbourne, Australia) to facilitate screening and full-text review [35]. We utilized a blinded, dual review process, with two levels of review: title/abstract followed by a full-text review. Conflicts were resolved by a third reviewer. Inclusion and exclusion criteria are presented in Table 2. We included digital interventions or technology descriptions from any country, provided that the article included technology that was, or could be, used to facilitate food assistance. A data extraction tool was developed in Covidence to extract the included articles’ title, first author last name, year of publication, data type, emergency setting country and city, food emergency cause, study aim, study design, type and purpose of intervention/tool, digital platform type, intervention methods, intended users, and government or non-government classification.

## 3. Results

**Figure 1:**
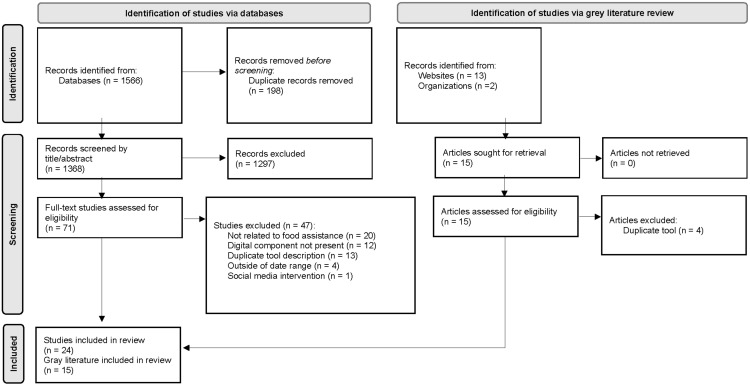
PRISMA flow diagram for new systematic reviews, which included searches of databases and grey literature. Adapted with permission from Ref [34].

The final search returned 1581 unique articles. Fifteen were from the grey literature and 1566 were peer-reviewed publications. Duplicates were removed (*n* = 198). A total of 1297 (88.3%) were excluded at the title/abstract screening stage. Eighty-six studies were selected for full-text review. Of these, 47 (64.4%) studies were excluded for the following reasons: 20 were not related to individual food assistance (42.6%), 13 described a duplicate tool (21.3%), 12 did not have a digital component (25.5%), 4 were outside of the date range (8.5%), and 1 described a social media campaign (2.1%). The PRISMA diagram is presented in Figure 1.

### 3.1. Descriptive Research Statistics

The final synthesis included 39 articles—15 from the grey literature and 24 from peer-reviewed articles (Table 3). The 39 studies described 39 unique tools. The digital tools examined in the studies were categorized into a primary platform: website (56.4%), smartphone application (20.5%), chatbot (5.1%), information network (2.6%), software program (2.6%), machine learning algorithm (2.6%), text messaging (2.6%), virtual learning (2.6%), and social media (2.6%). The types of data presented in the studies included: descriptive statistics (38.5%), text and opinion (38.5%), both quantitative and qualitative (10.3%), quantitative (7.7%), and models or code (5.1%). Articles that were only presenting text data described websites or mobile applications. The types of study designs presented in the articles included qualitative interview and focus group study (23.1%); website, application, or model development description (17.9%); cross-sectional study (7.7%); randomized control trial (5.1%); non-randomized experimental study (2.6%); case study (2.6%); and non-study (e.g., text and opinion; 41.0%).

**Figure 2:**
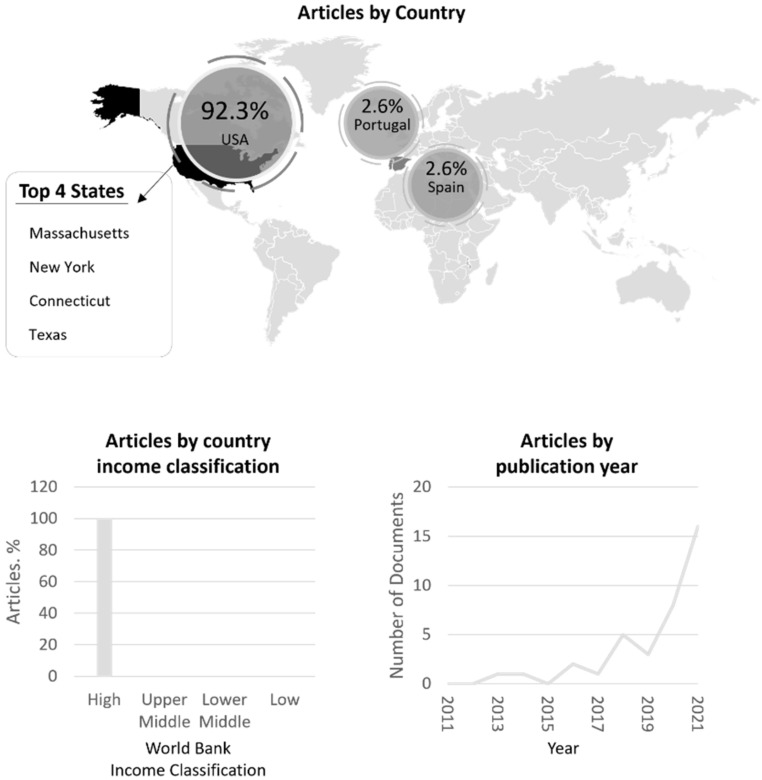
Article characteristics by setting, income status, and publication year.

Over 80% percent (84.6%) of the 39 articles were published in the last five years (i.e., 2016–2021) (Figure 2). The year with the highest volume of publications was 2021 (41.0%), followed by 2020 (20.5%). There were no studies published in 2015, 2014, 2012, or 2011. Two of the articles did not have a date specified (5.2%). Only three studies were located outside of the United States (in Spain and Portugal), and one was not specified. All of the other (93.2% of the studies) were U.S.-based. Regarding U.S.-based study settings, the top five states were Massachusetts (11.1%), New York (11.1%), Connecticut (8.3%), Tennessee (8.3%), Texas (8.3%), and California (5.6%). States with one study setting included: Colorado, Florida, Georgia, Illinois, Indiana, Michigan, Montana, New Jersey, New Mexico, Virginia, Washington, Washington, D.C., and West Virginia. Four studies were located within the United States but included national data collection or the exact location was not specified.

All of the included articles had clearly and intentionally specified the intended users of the various tools. The most frequent intended users were food pantry managers, staff, volunteers and/or clients (41.0%). Other users and/or intended beneficiaries included food-insecure individuals (general, 20.5%), WIC participants (15.4%), SNAP users (10.3%), managers and/or staff (10.3%), and policymakers (2.6%).

### 3.2. Desired versus Implemented Features in Digital Food Assistance Tools

This review identified five areas of desired features for food assistance management. ‘Desired feature’ refers to any features that the study author mentioned as important to digital food assistance within the study (e.g., typically within the introduction or discussion sections) but were not necessarily developed within the described tool. ‘Implemented feature’ refers to, conversely, desired features that are currently being implemented and included in the digital tool. These five areas included (1) food acquisition (donation management, donor engagement, fundraising, inventory management); (2) volunteer/staff management (recruitment, communication, scheduling, training, education); (3) client services (ordering, choice, enrollment, tracking, education, communications); (4) reporting (data collection and analysis, report maker, export report); and (5) emergency preparedness (training, protocols). The literature explicitly describes 19 specific features within these areas that are desired for food assistance digital tools, i.e., website or smartphone application (Supplementary Table S1).

These 19 desired features include: volunteer management; inventory management; client tracking; online ordering; staff training; client training; emergency preparedness training and protocols; client choice; nutrition guidelines and education for clients and staff; report exporting; data analytics; volunteer recruitment; communications; social media sharing; prediction of food insecurity events; distribution event calendar; pantry locator; donations tracker; and grants tracker. Table 4 shows the number of features per tool, and Table 5 shows the number of tools by feature.

The Food Pantry Helper and Pantri websites had the highest number of features (*n* = 9). Thirty of the tools had one or two features. This included, for example, the Volgistics website, which supports volunteer management, e.g., recruitment, scheduling, and messaging. WIC Shopper, on the other hand, singularly supported online grocery shopping with the Electronic Benefit Transfer (EBT) card for SNAP and WIC participants.

The most frequently included feature in the digital tools was online grocery ordering (e.g., using SNAP benefits to purchase free or discounted foods online; number of tools = 14), followed by inventory management (number of tools = 12), client tracking (clients could refer to WIC participants, SNAP users, and/or food pantry users; number of tools = 11), volunteer management (number of tools = 9), nutrition guidelines/education (number of tools = 8), and report exporting (number of tools = 8). The features that were identified as important by the review but were not incorporated in the reviewed tools included client training, emergency preparedness, and social media sharing.

### 3.3. Characteristics of Peer-Reviewed Digital Tools for Food Assistance

As the grey literature articles were solely text and opinion and not actual studies, the peer reviewed literature subset of articles was analyzed separately to determine study and tool characteristics. In the peer-reviewed literature, studies focused on researching online ordering (*n* = 5), training and education (*n* = 5), pantry inventory management (*n* = 4), *communications* (*n* = 4), improving public health interventions (*n* = 3), pantry volunteer management (*n* = 2), and applying to online *SNAP or WIC* (*n* = 1) (Table 6). Many of these tools were developed and implemented by academics (*n* = 11), followed by academic-local partners (*n* = 6).

Online ordering refers to the ability of clients of food pantries, SNAP users, or WIC participants to do online grocery shopping with or without their SNAP benefits [30,36,37,41,45,46,47,48,49,52,54,59]. The developers of these tools included academics, academic-WIC partners, academic-USDA partners, and academic-non-profit organization partners. The intended users and beneficiaries included food-insecure individuals, WIC participants, SNAP users, and food pantry managers. The top challenges reported in rank order were (1) accessing the population; (2) mistrust of online shopping systems; and (3) lack of knowledge regarding healthy foods.

When the primary purpose of the tool was training and education, study personnel aimed to develop online materials or interactive courses for staff, volunteers, or food-insecure individuals [38,44,66,68]. The primary developers of these tools were academics (*n* = 4) and medical professionals (*n* = 1). The intended beneficiaries or users included SNAP users, food-insecure individuals (general), and WIC participants. The top three reported challenges were (1) lack of cultural acceptability, (2) learning curve of using a phone for educational purposes, and (3) low or waning participation over time.

Studies implementing or evaluating inventory management tools involved tracking products going in and out of food pantries or banks, both from the donor- and client-perspectives [25,36,40,43,48,50,54,55,64,65,69,74]. Two of these tools were developed by academics, and two were developed by food pantries. The intended users or beneficiaries included food-insecure individuals (general), food pantry staff or volunteers, and donors. The top three reported challenges in rank order were (1) poor funding for inventory management outside of emergency situations, (2) lack of data from donor organizations, and (3) lack of funding or foresight for supplier shipping costs.

Communications tools described by these studies refer to messaging between pantries and clients or volunteers [37,42,51,63]. These were developed by academics (*n* = 2), a non-profit-organization (*n* = 1), and an academic–community partnership (*n* = 1). Food-insecure individuals (general), SNAP users, and WIC participants were the intended beneficiaries and users. Frequently reported challenges in rank order included (1) lack of ability to reach vulnerable populations in general, (2) concerns about creating more or redundant work for staff, and (3) lack of preparedness for emergency situations.

Three studies aimed to create digital tools to improve the use and acceptability of public health interventions [50,53,73]. This included combining online grocery shopping with nutrition education to improve healthy food options and consumption at food pantries. These tools were intended for policymakers, stakeholders, managers, WIC participants, and food pantry clients. All studies reported a challenge with the need to continuously update their services to meet the needs of new users.

For two studies, the primary purpose of the tools was volunteer management [60,64]. This included scheduling, communications, training, onboarding, and recruitment of volunteers on behalf of food pantries. The main developers of these two tools were a food pantry and a food pantry–academic partnership. The intended beneficiaries of this tool were food pantry volunteers, managers, staff, and donors. The top reported challenge was staff fatigue from using multiple online tools for pantry management.

Finally, one study evaluated online applications for new SNAP or WIC participants [63]. This entailed food-insecure individuals applying online to SNAP or WIC via a website and a chatbot. This study was developed through an academic–WIC partnership to benefit WIC participants. The top challenges with this tool were (1) concerns about fraud and churn rate (the annual percentage rate at which customers stop subscribing to a service [75]) and (2) discrimination in the online application system and review process.

## 4. Discussion

While there are many reviews on mHealth strategies to improve public health [76], this is the first to explore the digital tools that exist to support food assistance programs. Our findings add to the literature by defining the scope of research and development and identifying gaps in current technology for food assistance. Surprisingly, over 90% of the studies were based in the United States, and 100% of them were in developed countries. Overall, this review defines a list of desired application features that are important for online food assistance, including volunteer and inventory management, communications, online ordering, promoting healthier pantry options, and client tracking. From this list, this review tallies the current state of the development and implementation of these features. It also categorizes the types of websites and mobile applications in use and identifies gaps in related publications, use of modern technologies, and breadth of coverage of locations (i.e., no studies were conducted in low-income countries).

Of timely and ongoing relevance to food-insecurity threats to population health, our review identified five areas currently being implemented online: (1) food acquisition (donation management, donor engagement, fundraising, inventory management); (2) volunteer/staff management (recruitment, communication, scheduling, training, education); (3) client services (ordering, choice, enrollment, tracking, education, communications); (4) reporting (data collection and analysis, report maker, export report); and (5) emergency preparedness (training, protocols). While this review identified 39 unique digital tools, these tools cover a small fraction of the 19 desired features and are not being widely implemented. Most were highly specialized and employed 1–2 features per tool (*n* = 30). For example, Pantri and Food Pantry Helper had the most features (*n* = 9) but are still missing 10 features that were identified as important.

An important theme identified was the need to include healthy eating education with online shopping to improve nutritional outcomes for food-insecure individuals. The identified literature points to a general agreement that providing equitable access to online, free-to-discounted shopping is not adequate to improve health outcomes; nutrition education is important. Findings from this review suggest that the main avenue for achieving this goal is to build websites with dual features of online ordering with pop-ups and green light–red light systems, such as SWAP (Supporting Wellness at Pantries) [77]. SWAP is a stoplight nutrition system that ranks food based on levels of saturated fat, sodium, and sugars because these nutrients are linked with an increased risk of chronic diseases. Some websites have separate nutrition trainings, while others have pop-up messages or messages underneath food items regarding nutritional content. While the developers of these tools, mostly academics, have stated the importance of education to support healthier online purchasing, these presented education tools were not evaluated by appropriate public health professionals (i.e., nutrition experts, outcome evaluation experts). Some studies incorporated aspects of the user-centered design framework to verify if the educational components were culturally acceptable or user-friendly. Indeed, Rogero et al. reported a lack of cultural acceptance to be a major challenge to implementing education with online shopping [46]. None of the studies followed the entire user-center design framework, however. Additionally, the tools presented are all in early development phases, and their efficacy has not yet been reported.

Regarding the provision of food supply from donors to food pantries, while online ordering was a major theme in the corpus, there were no studies reporting its use in food pantries. Studies authored by pantries did underscore the importance of client choice (in which clients can select their own items), but none reported the use of a digital tool to do so. Only two tools found through the grey literature search, *Flemington Area Food Pantry* and Lakeview Pantry Online Market, provide online forms for clients to communicate food preferences in their next pantry order [47,54]. Client choice, or the ability for food pantry users to select their own food, has been shown to be important for achieving food security and health outcomes. It supports important outcomes, such as ‘client dignity, agency, reducing food waste, and addressing individual dietary needs’ [78,79]. Digital apps with client choice features offer a currently underexplored opportunity to improve food assistance.

The digital tools for pantries focused on three aspects of this supply chain: food bank acquisition of food from donors, manager education to acquire healthier food for food pantries from banks, and food pantry management. In the identified literature from this review, the food pantry management aspect focused primarily on only two elements: namely, volunteer and inventory management. Emergency preparedness, client training, and online training were often stated as desired but not included in the online strategies.

While the 39 tools varied in feature implementation, most of the studies reviewed are only in the early development and implementation phases. Additionally, they exhibit tool descriptions and opinions, rather than quantitative and/or qualitative data. This lack of effectiveness and impact data undermines the ability to predict the successes and challenges of more wide-scale implementation of these strategies. Future work must therefore focus on impact and effectiveness studies as well as wide-scale implementation in food assistance programs.

Additionally, the reviewed studies are limited in location, with over 90% of studies situated in the United States. This result is of noteworthy concern, as food security is a global issue, and other countries have programs on individual food assistance efforts. One application from outside the U.S. was the OLIO food sharing app from the United Kingdom [80]. Users post photos of surplus food in the app and those in need (food-insecure or not) are able to connect and receive these items. It is possible that small programs in other countries are either not developing digital tools or not publicizing these tools for individual food assistance. Additional qualitative research, e.g., based on in-depth interviews, is needed in other countries to determine the state of digital tools for food assistance.

Of benefit, these articles in this literature review highlighted a variety of digital features that food assistance programs are interested in implementing. The current state of digital tools, however, does not meet these needs. The digital tools reviewed were limited in features. Most were highly specialized websites employing 1–2 features. Pantri and Food Pantry Helper had the most features (*n* = 9) but are still missing 10 features that were identified as important. The missing features were client choice, client training, emergency preparedness, donations and grants trackers, online ordering, pantry locator, social media sharing, staff training, and volunteer recruitment. Creating one user-friendly platform for all features is extremely important to food assistance managers to avoid the commonly stated challenges (not wanting to use multiple platforms, multiple platforms leading to inefficient use of time and money, too much training to learn multiple platforms, needing to be on the phone and not a desktop).

A gap between desired and actual features included those for emergency response. The COVID-19 pandemic has shown that food assistance programs were not prepared for emergency settings. Furthermore, none of the reviewed digital tools included emergency response features. This could have included emergency operations protocols, contact information, online services, and emergency response training for staff and clients [81,82].

A potential limitation of any new technology is its acceptance and usability by the target users. Indeed, a frequently cited concern in the findings was regarding uncertainty if these tools will be sustainable over the long term, with regard to both funding and participation. These concerns can be addressed by developing the digital strategies in collaboration with the intended users and beneficiaries [83,84]. There are two frameworks commonly used to improve this in mHealth interventions: Community-Based Participatory Research and User-Centered Design [85,86,87]. Both of these frameworks include the end-user (e.g., food pantry clients) within the intervention development process, ensuring that the tools meet the needs of intended users [88]. In addition, stakeholder management and analysis frameworks provide valuable protocols for improving acceptability and usability [3,4]. Despite these frameworks, only 12 studies were performed in partnership with or led by food assistance programs. None of the studies or tools presented in this review included the utilization of a user-centered design process during the development of the digital strategy. Two of the online shopping tools identified in this literature review characterized user opinions regarding online shopping. While this line of questioning is one aspect of user-centered design, it is very preliminary and does not adequately involve users in the development process. There is, accordingly, an urgent need for more studies that prominently integrate evidence-informed best practices of user-centered design to enhance food security using digital technology.

Food assistance stakeholders may use this review to identify tools to incorporate in their programming and serve their communities more effectively. Certainly, as the current pandemic—and future disasters—continue to force programs towards paperless and contactless services, there will be a higher and higher need and demand for these digital tools [24]. The food assistance sector must speed up its movement towards the modern digital age [32].

### Limitations

There were several limitations to this study that may have impacted the results. While the search string was built to capture the entire corpus that could address our research questions, some studies may have been overlooked. Related, the definitions of ‘food assistance’ and ‘digital tools’ are complicated, and therefore, there are many synonyms to describe these concepts. Our search may not have included all variations of these concepts and therefore missed critical studies. The search also only included articles in English; this may have led to the exclusion of important, non-English language studies. While our grey literature search included several key governmental and non-governmental search engines and websites, the search may not have found tools within local pantries or organizations that have not formally published in the major databases used in our research. Lastly, the review did not consider the quality of the studies themselves.

Despite these limitations, this study highlights critical gaps in digital tools for food assistance and characterizes the scope of the extant literature through a food assistance technological lens amidst an ever-broadening array of threats to food security.

## 5. Conclusions

The COVID-19 pandemic revealed the importance of having online food assistance tools to meet the growing needs of food-insecure individuals. Food assistance programs may be more willing—or, indeed, forced by necessity—to adopt novel technological solutions to address these evolving challenges. Providing timely support for food assistance programs to switch from in-person or paper-based systems to digital platforms should be the subject of future work. While this review identified 39 tools, all are in the early development and/or implementation stages. Review findings highlight an overall lack of these tools, an absence of user-centered design in their development, and a critical need for research on their effectiveness globally. Further analysis and testing of current digital tool usage and interventions examining the health and food security impacts of such tools should be explored in future studies, including in the context of pandemics, where digital tools allow for help from a distance. Furthermore, effectiveness and impact studies on these digital tools must be prioritized. This will ensure that the best and most cost-effective strategies are advancing in the face of an ever-expanding array of emergent threats to food security.

## Figures and Tables

**Table 1:** Databases included, search Terms utilized, and search results by database.

Databases	
Bibliographic Databases	PubMed, Embase, Scopus, Web of Science, PsychInfo, Cinhal
Grey Literature	Google, Science.gov, Worldwidescience.org, World Food Programme Website, U.S. Agency for International Development Website, UNICEF Website, Feeding America Website
Key Search Terms *	
Example Terms	Application; App; Smartphone; Mobile; Website; Online, Desktop; Computer; Web; Internet Food Assistance; Food Supply; Food Bank; Food Security
Search Results	
Pubmed	198
Embase	170
Scopus	774
Web of Science	245
PsychInfo	115
Cinhal	260

* The key search terms were edited/formatted for each database.

**Table 2:** Article inclusion and exclusion criteria.

Field	Inclusion Criteria	Exclusion Criteria
Publication Date Range	2011–2021	Outside of date range
Technology Type	Digital applications (website, desktop, mobile, smartphone)	Non-digital
Technology Purpose	Individual Food Assistance	Agriculture sector food security issue Online SNAP application Online nutrition education (not intended for food-insecure individuals explicitly)
Population	WIC Participants SNAP Users Food Pantry Users Food-Insecure Individuals	Not explicitly food-insecure individuals
Language	English Only	Non-English language

**Table 3:** Characteristics of reviewed articles (*n* = 39).

Study ID	Year	Setting	Study Design	Types of Data	Tool Type	Tool Purpose	Tool Name
Analytic Solutions, Inc. [36]	2021	United States, Massachusetts, Boston	Text and opinion	App description	Website	Online ordering; inventory management; reporting	Smart Choice Pantry
Bertrand et al. [37]	2021	United States, Washington, D.C.	Cross sectional study	Quantitative	Social media	Communications	Not specified
Biediger-Friedman et al. [38]	2016	United States, Texas, Regional	Qualitative research	Qualitative	Smartphone app	Training/ education	my WIC Family
Biediger-Friedman, et al. [39]	2019	United States, Texas, Statewide	Qualitative research	Qualitative	Chatbot	Communications	Maya, the Texas WIC Chatbot
Blackmon et al. [40]	2021	United States, California, Los Angeles	Model/code development	Models/code only	Software program	Inventory management	Decision Support System
Boston Medical Center Vital Village Network Data Workgroup [41]	2018	United States, Massachusetts, Boston	Text and opinion	App description	Smartphone app	Pantry locator; food resource review	Abundance Boston
Carvalho et al. [42]	2020	Portugal, Braga District	Case study, series, or report	Quantitative and qualitative	Information network	Communications	Emergency Food Network
Cedar Mountain Software [43]	2021	Untied States, Montana, Missoula	Text and opinion	App description	Website	Inventory management; appointment scheduling; online customer portal; client management; custom reporting; visits; registration; consultations	Pantry Soft
Clarke et al. [44]	2018	United States, California, Los Angeles	Randomized controlled trial	Quantitative	Smartphone app	Training/ education	VeggieBook
Coffino et al. [45]	2020	United States, New York	Randomized controlled trial	Quantitative	Website	Online ordering	Not specified
Connecticut Food Bank [46]	2020	United States, Connecticut, Statewide	Text and opinion	App description	Website	Communications	CT Food Bank Mobile Pantry Schedule
Flemington Area Food Pantry [47]	2021	United States, New Jersey, Flemington	Text and opinion	App description	Website	Online ordering	Flemington Food Pantry Ordering
Food Pantry Helper [48]	2021	United States, New York, Saratoga Springs	Text and opinion	App description		Client tracking; inventory management; grant tracking; donations tracking; volunteer management; communications; reporting	Food Pantry Helper
Food Pantry Manager [49]	2021	Not specified	Text and opinion	App description	Website	Reporting; dashboard analytics; volunteer management; online ordering; client/payment tracking; 24/7 support	Food Pantry Manager
Hamad et al. [50]	2019	United States, National	Model/code development	Quantitative and qualitative	Machine learning	Improve targeted interventions	Not specified
Herron et al. [51]	2021	United States	Qualitative research	Qualitative	Text messaging campaign	Communications	Not specified
Hull et al. [52]	2017	United States, Tennessee, Nashville	Qualitative research	Qualitative	Unclear? Not stated?	WIC online shopping and nutrition education	CHEW
Jia et al. [53]	2021	United States, Massachusetts, Boston	Qualitative research	Qualitative	Website	Improve healthy food options at pantries	Healthy Pantry Program
Lakeview Pantry [54]	2021	United States, Illinois, Chicago	Text and opinion	App description	Website	Client registration; client tracking; inventory management; online ordering	Lakeview Pantry Online Martket
Link2Feed [55]	2013–2021	United States, Michigan, Detroit	Text and opinion	App description	Website	Volunteer management; inventory management; client self enrollment; client intake; reporting; dashboard analytics	Link2Feed
Martin et al. [56]	2020	United States, Connecticut, Bloomfield	Non-randomized experimental study	Quantitative and qualitative	Website	Online ordering	Not specified
Mid-North Food Pantry [57]	2021	United States, Indiana, Indianapolis	Text and opinion	App description	Website	Use signup.com to manage volunteers	Mid-North Food Pantry
Moguel et al. [58]	2020	Spain	Model/code development	Models/code only	Smartphone app	Inventory management	YourPantry
New York City Food Assistance Collaborative [59]	2018	United States, New York, New York City	Text and opinion	App description	Website	Reservation system for food pantries; client tracking; reporting; pantry info	Plentiful App
Pichardo et al. [60]	2016	United States, New York, Pleasantville	App/website development description	Qualitative	Smartphone app	Volunteer management	NEED2FEED
Rogus et al. [61]	2020	United States, New Mexico, Las Cruces	Cross sectional study	Qualitative	Website	Online shopping	Not specified
Schoch et al. [62]	2019	United States, Washington, Tacoma	App/website development description	Description of app development	Smartphone app	Online ordering	The Food Locker
Scott et al. [63]	2020	United States, Texas, Statewide	Qualitative research	Qualitative	ChatBot	SNAP/WIC online application	Not specified
Sewald et al. [64]	2018	United States, Colorado, Boulder	App/website development description	Qualitative	Website	Volunteer and inventory management	Food Rescue Robot
Solutions by Solutions, Inc. [65]	2013	United States, Florida, Naples	Text and opinion	App description	Website	Inventory management; client tracking; reporting	Pantry Worx
Stotz et al. [66]	2018	United States	Qualitative research	Qualitative	Website	Training/ education	Food eTalk
The Online Food Pantry [67]	2021	United States, Georgia, Hampton	Text and opinion	App description	Website	Client tracking; donor tracking; inventory management; online ordering; volunteer management	The Online Food Pantry
Traub et al. [68]	2020	United States, Connecticut, Statewide	Qualitative research	Qualitative	Website	Training/ education	SNAP4CT
Ufot et al. [69]	2021	United States, North Carolina, Greensboro	App/website development description	Qualitative	Smartphone app	Inventory management	Not specified
Volgistics [70]	2004–2021	United States, Not specified	Text and opinion	App description	Website	Volunteer management	Volgistics
Weinstein et al. [71]	2021	United States, Massachusetts, Boston	Text and opinion	Qualitative	Virtual learning	Communications	Nourishing Our Community
Zhang et al. [72]	2021	United States, West Virginia, Statewide	Cross sectional study	Quantitative and Qualitative	Smartphone app	Online shopping	WICShopper
Zimmer et al. [73]	2021	United States, Tennessee, Regional	Qualitative research	Qualitative	Website	Online ordering	Not specified
San Bernadino Food Pantry [74]	2021	United States, California, San Bernadino	Text and opinion	App description	Website	Volunteer management; pantry events calendar; inventory tracking; form Builder; nutrition Guidelines; client tracking; data analytics; export Report; machine learning-predict pantry trends; ongoing support	Pantri

**Table 4:** Number of features per tool.

Tool Name	Number of Features	Percentage of Features
Food Pantry Helper	9	47.4
Pantri	9	47.4
Food Pantry Manager	5	26.3
Link2Feed	5	26.3
The Online Food Pantry	5	26.3
Food Rescue Robot	4	21.1
Pantry Soft	4	21.1
Lakeview Pantry Online Market	4	15.8
Pantry Worx	3	15.8
Plentiful App	3	15.8
Smart Choice Pantry	3	15.8
CHEW	2	10.5
CT Food Bank Mobile Pantry Schedule	2	10.5
Food eTalk	2	10.5
my WIC Family	2	10.5
SNAP4CT	2	10.5
VeggieBook	2	10.5
Decision Support System	1	5.3
Emergency Food Network	1	5.3
Flemington Food Pantry Ordering	1	5.3
Healthy Pantry Program	1	5.3
Maya, the Texas WIC ChatBot	1	5.3
Midnorth Food Pantry	1	5.3
NEED2FEED	1	5.3
Bertrand et al.	1	5.3
Coffiino et al.	1	5.3
Hamad et al.	1	5.3
Herron et al.	1	5.3
Martin et al.	1	5.3
Rogus et al.	1	5.3
Scott et al.	1	5.3
Ufot et al.	1	5.3
Zimmer et al.	1	5.3
Nourishing Our Community	1	5.3
The Food Locker	1	5.3
Volgistics	1	5.3
WICShopper	1	5.3
YourPantry	1	5.3
Abundance Boston	1	5.3

**Table 5:** Number of tools per feature.

Feature Name	Number of Tools	Percentage of Tools
Online Ordering	14	35.9
Inventory Management	12	30.8
Client Tracking	11	28.2
Volunteer Management	9	23.1
Nutrition Guidelines/Education	8	20.5
Report Exporting	8	20.5
Communications	6	15.4
Staff Training	4	10.3
Data Analytics	4	10.3
Pantry Locator	3	7.7
Donations Tracker	3	7.7
Distribution Event Calendar	2	5.1
Client Choice	2	5.1
Volunteer Recruitment	1	2.6
Prediction of Food Insecurity	1	2.6
Grants Tracker	1	2.6
Client Training	0	0.0
Emergency Preparedness	0	0.0
Social Media Sharing	0	0.0

**Table 6:** Digital tools for food assistance peer-reviewed research summary.

Primary Tool Purpose	Description	Number of Articles	Tool Developers	Intended Beneficiaries and/or Users	Top Challenges
Online Ordering	Clients of food pantries, SNAP users, and/or WIC participants can shop online with or without their SNAP benefits	5	Academics (2); academic–WIC partnership (2); academic–USDA partnership (1); non-profit academic partnership (1)	Food-insecure individuals (2); WIC participants (2), SNAP users (1), managers/staff (1)	(1) Accessing the population; (2) mistrust; (3) knowledge of healthy foods; (4) little known about perspectives of WIC participants on online grocery shopping; (5) low participation; (6) social barriers, e.g., embarrassment
Training/Education	Online materials and/or interactive courses for staff, volunteers, and/or food-insecure individuals	5	Academics (4), medical professionals (1)	SNAP users (2), food-insecure individuals (2), WIC participants (1)	(1) Low participation; (2) cultural acceptability; low-income user focus; (3) learning curve in using phones
Inventory Management	Tracking products going in and out of food pantries and/or banks, both from donor side and client side	4	Academics (2), food Pantry (2)	Food-insecure individuals (2), managers/Staff (1); food pantry volunteers, managers, staff, donors (1)	(1) Elderly access to foods; (2) supplier shipping costs not considered in system; (3) no economic incentives for distributors to collaborate outside of emergency aid situations.
Communications	Messaging between pantries and clients and/or volunteers	4	Academics (2), non-Profit-organization (1), academic–community partnership (1)	Food-insecure individuals (2), SNAP users (1), WIC participants (1)	(1) Hard to reach populations via social media and other communications methods, (2) worried about creating more work for WIC staff; (3) cultural acceptability; (4) mistrust
Improve use and acceptability of public health interventions	Online shopping plus education to improve healthy food options at food pantries	3	Academics, hospital, food bank, community	Policymakers/stakeholders; managers/staff. WIC participants, food pantry clients	Update services to meet needs of newer users
Volunteer Management	Scheduling, communications, training, onboarding, recruitment	2	Non-profit organization (1); academic–pantry partnership	Volunteers, managers, staff, donors (2)	(1) Logistics and volunteer tracking
SNAP/WIC Online Application	Food-insecure individuals apply to SNAP or WIC via online website	1	Academic–WIC partnership (1)	WIC participants	(1) Concerns about fraud and churn; (2) concerns about discrimination

## Data Availability

Not applicable.

## Supplementary Materials

The following supporting information can be downloaded at: https://www.mdpi.com/article/10.3390/ijerph19031328/s1, Table S1: Search term example: SCOPUS; Table S2: Tools by implemented features.

## Abbreviations

U.S. (United States), PRISMA (Preferred Reporting Items for Systematic Reviews and Meta-Analyses) Checklist), SNAP (Supplemental Nutrition Assistance Program), WIC (Special Supplemental Nutrition Program for Women, Infants, and Children), SWAP (Supporting Wellness at Pantries).

## References

[1] WHO WHO|Monitoring and Evaluating Digital Health Interventions. http://www.who.int/reproductivehealth/publications/mhealth/digital-health-interventions/en/.

[2] Hatanaka M. (2020). Beyond consuming ethically? Food citizens, governance, and sustainability. J. Rural Stud..

[3] Chen S.C.-I., Liu C., Wang Z., McAdam R., Brennan M., Davey S., Cheng T.Y., Hu R.D. (2020). How Geographical Isolation and Aging in Place Can Be Accommodated through Connected Health Stakeholder Management: Qualitative Study with Focus Groups. J. Med. Internet Res..

[4] Scott M.K., Gutuskey L., Zwemer T., Gallington K. (2020). Farmers Market Food Navigator Program: Key Stakeholder Perceptions and Program Outcomes. Health Promot. Pract..

[5] Rose T., Barker M., Jacob C.M., Morrison L., Lawrence W., Strömmer S., Vogel C., Woods-Townsend K., Farrell D., Inskip H. (2017). A Systematic Review of Digital Interventions for Improving the Diet and Physical Activity Behaviors of Adolescents. J. Adolesc. Health.

[6] Baskerville N.B., Struik L.L., Guindon G.E., Norman G.D., Whittaker R., Burns C., Hammond D., Dash D., Brown K.S. (2018). Effect of a Mobile Phone Intervention on Quitting Smoking in a Young Adult Population of Smokers: Randomized Controlled Trial. JMIR mHealth uHealth.

[7] Jane M., Hagger M., Foster J., Ho S., Kane R., Pal S. (2017). Effects of a weight management program delivered by social media on weight and metabolic syndrome risk factors in overweight and obese adults: A randomised controlled trial. PLoS ONE.

[8] Willis E.A., Szabo-Reed A., Ptomey L., Steger F.L., Honas J.J., Washburn R.A., Donnelly J.E. (2016). Do weight management interventions delivered by online social networks effectively improve body weight, body composition, and chronic disease risk factors? A systematic review. J. Telemed. Telecare.

[9] Ashrafian H., Toma T., Harling L., Kerr K., Athanasiou T., Darzi A. (2014). Social Networking Strategies That Aim to Reduce Obesity Have Achieved Significant although Modest Results. Health Aff..

[10] Beleigoli A.M., Andrade A.Q., Cançado A.G., Paulo M.N., Diniz M.D.F.H., Ribeiro A.L. (2019). Web-Based Digital Health Interventions for Weight Loss and Lifestyle Habit Changes in Overweight and Obese Adults: Systematic Review and Meta-Analysis. J. Med. Internet Res..

[11] Dunn E.E., Gainforth H.L., Robertson-Wilson J.E. (2018). Behavior change techniques in mobile applications for sedentary behavior. Digit. Health.

[12] Wu J., Guo S., Huang H., Liu W., Xiang Y. (2018). Information and Communications Technologies for Sustainable Development Goals: State-of-the-Art, Needs and Perspectives. IEEE Commun. Surv. Tutorials.

[13] Olu O., Muneene D., Bataringaya J.E., Nahimana M.-R., Ba H., Turgeon Y., Karamagi H., Dovlo D. (2019). How Can Digital Health Technologies Contribute to Sustainable Attainment of Universal Health Coverage in Africa? A Perspective. Front. Public Health.

[14] Ibeneme S., Ukor N., Ongom M., Dasa T., Muneene D., Okeibunor J. (2020). Strengthening capacities among digital health leaders for the development and implementation of national digital health programs in Nigeria—PubMed. BMC Proceedings.

[15] Deacon A.J., Edirippulige S. (2015). Using mobile technology to motivate adolescents with type 1 diabetes mellitus: A systematic review of recent literature. J. Telemed. Telecare.

[16] Castensøe-Seidenfaden P., Husted G.R., Jensen A.K., Hommel E., Olsen B., Pedersen-Bjergaard U., Kensing F., Teilmann G. (2018). Testing a Smartphone App (Young with Diabetes) to Improve Self-Management of Diabetes over 12 Months: Randomized Controlled Trial. JMIR mHealth uHealth.

[17] Willis E.A., Szabo-Reed A.N., Ptomey L., Steger F.L., Honas J.J., Al-Hihi E.M., Lee R., Lee J., Oh Y., Washburn R.A. (2017). Distance learning strategies for weight management utilizing online social networks versus group phone conference call. Obes. Sci. Pract..

[18] Sepah S.C., Jiang L., Peters A.L. (2014). Translating the Diabetes Prevention Program into an Online Social Network: Validation against CDC Standards. Diabetes Educ..

[19] Dholakia U., Tsabar G. (2011). A Startup’s Experience with Running a Groupon Promotion. https://papers.ssrn.com/sol3/papers.cfm?abstract_id=1828003.

[20] Food Assistance Programs|Nutrition.gov. https://www.nutrition.gov/topics/food-assistance-programs.

[21] Supplemental Nutrition Assistance Program (SNAP)|Food and Nutrition Service. https://www.fns.usda.gov/snap/supplemental-nutrition-assistance-program.

[22] Special Supplemental Nutrition Program for Women, Infants, and Children (WIC)|Food and Nutrition Service. https://www.fns.usda.gov/wic.

[23] Coleman-Jensen A., Gregory C., Singh A. (2014). Household Food Security in the United States.

[24] Niles M.T., Bertmann F., Belarmino E.H., Wentworth T., Biehl E., Neff R. (2020). The Early Food Insecurity Impacts of COVID-19. Nutrients.

[25] Hernandez D.C., Holtzclaw L.E. (2021). Commentary: The Impact of the COVID-19 Pandemic and the Economic Recession on Food Insecurity: Short- and Long-term Recommendations to Assist Families and Communities. Fam. Community Health.

[26] Dzhanova Y. (2020). Food Banks Are Closing and Losing Their Workforce Because of the Coronavirus. CNBC. https://www.cnbc.com/2020/04/28/coronavirus-food-banks-are-closing-and-losing-their-workforce.html.

[27] Kulish N. (2020). Food Banks Are Overrun, as Coronavirus Surges Demand. The New York Times.

[28] Al-Ramahi M., Elnoshokaty A., El-Gayar O., Nasralah T., Wahbeh A. (2021). Public Discourse against Masks in the COVID-19 Era: Infodemiology Study of Twitter Data. JMIR Public Health Surveill..

[29] Chen E., Lerman K., Ferrara E. (2020). Tracking Social Media Discourse About the COVID-19 Pandemic: Development of a Public Coronavirus Twitter Data Set. JMIR Public Health Surveill..

[30] Pascual-Ferrá P., Alperstein N., Barnett D.J. (2020). Social Network Analysis of COVID-19 Public Discourse on Twitter: Implications for Risk Communication. Disaster Med. Public Health Prep..

[31] Pascual-Ferrá P., Alperstein N., Barnett D.J. (2021). A Multi-platform Approach to Monitoring Negative Dominance for COVID-19 Vaccine-Related Information Online. Disaster Med. Public Health Prep..

[32] Martin N., Sundermeir S., Barnett D., Van Dongen E., Rosman L., Rosenblum A., Gittelsohn J. (2021). Digital Strategies to Improve Food Assistance in Disasters: A Scoping Review. Disaster Med. Public Health Prep..

[33] Barnett D.J., Rosenblum A.J., Strauss-Riggs K., Kirsch T.D. (2020). Readying for a Post–COVID-19 World: The Case for Concurrent Pandemic Disaster Response and Recovery Efforts in Public Health. J. Public Health Manag. Pract..

[34] Page M.J., McKenzie J.E., Bossuyt P.M., Boutron I., Hoffmann T.C., Mulrow C.D., Shamseer L., Tetzlaff J.M., Akl E.A., Brennan S.E. (2021). The PRISMA 2020 statement: An updated guideline for reporting systematic reviews. J. Clin. Epidemiol..

[35] (2016). Covidence—Better Systematic Review Management. https://www.covidence.org/.

[36] Smart Choice—Online Food Pantry Inventory and Shopping Software United States. https://www.smartchoicepantry.com.

[37] Bertrand A., Hawkins M., Cotter E.W., Banzon D., Snelling A. (2021). Interest in Receiving Nutrition Information through Social Media among Food-Security Program Participants in Washington, DC. Prev. Chronic Dis..

[38] Biediger-Friedman L., Crixell S.H., Silva M., Markides B.R., Smith K.S. (2016). User-centered Design of a Texas WIC App: A Focus Group Investigation. Am. J. Health Behav..

[39] Biediger-Friedman L., Crixell S., Scott C., Markides B.R. (2019). User-Centered Design of a Texas WIC ChatBot: Formative Investigation.

[40] Blackmon L., Chan R., Carbral O., Chintapally G., Dhara S., Felix P., Jagdish A., Konakalla S., Labana J., McIlvain J. (2021). Rapid Development of a Decision Support System to Alleviate Food Insecurity at the Los Angeles Regional Food Bank amid the COVID-19 Pandemic. Prod. Oper. Manag..

[41] Abundance, Home. https://www.abundanceboston.com.

[42] Carvalho J., Sousa R.D. Exploring the case of a local food bank to understand information technology use in government information networks. Proceedings of the 13th International Conference on Theory and Practice of Electronic Governance.

[43] Pantry SoftFood Pantry Intake & Reporting Solution. PantrySOFTTM. https://www.pantrysoft.com/.

[44] Clarke P., Evans S.H., Neffa-Creech D. (2018). Mobile app increases vegetable-based preparations by low-income household cooks: A randomized controlled trial. Public Health Nutr..

[45] Coffino J.A., Udo T., Hormes J.M. (2020). Nudging while online grocery shopping: A randomized feasibility trial to enhance nutrition in individuals with food insecurity. Appetite.

[46] Mobile Pantry Schedule|Connecticut Food Bank. https://www.ctfoodbank.org/get-help/connecticut-food-banks-mobile-pantry-schedule/.

[47] Order. Flemington Food Pantry. https://flemingtonfoodpantry.org/order.

[48] Food Pantry Helper—Food Pantry Helper. https://www.foodpantryhelper.com/.

[49] Food Pantry Manager|Food Pantry Software for $15 a Month. https://www.foodpantrymanager.org/.

[50] Hamad R., Templeton Z.S., Schoemaker L., Zhao M., Bhattacharya J. (2019). Comparing demographic and health characteristics of new and existing SNAP recipients: Application of a machine learning algorithm. Am. J. Clin. Nutr..

[51] Herron B., Anthony D., Bell D., Cotto-Rivera E., Lee J.S. (2021). P92 Needs Assessment for a Mobile Text Messaging Intervention for SNAP-Eligible Adults. J. Nutr. Educ. Behav..

[52] Hull P., Emerson J.S., Quirk M.E., Canedo J.R., Jones J.L., Vylegzhanina V., Schmidt D.C., Mulvaney S.A., Beech B.M., Briley C. (2017). A Smartphone App for Families with Preschool-Aged Children in a Public Nutrition Program: Prototype Development and Beta-Testing. JMIR mHealth uHealth.

[53] Jia J., Caty R., Fiechtner L., Zack R., Thorndike A.N. (2021). Abstract MP19: Academic-Community Partnership to Evaluate the Effectiveness of the Greater Boston Food Bank’s Healthy Pantry Program. Circulation.

[54] Online Market|New Convenient Food Pickup Service. Lakeview Pantry. https://www.lakeviewpantry.org/get-food/onlinemarket/.

[55] Link2Feed Food Bank Pantry Software. Link2Feed. https://www.link2feed.com/.

[56] Martin K., Xu R., Schwartz M.B. (2020). Food pantries select healthier foods after nutrition information is available on their food bank’s ordering platform. Public Health Nutr..

[57] Mid-North Food Pantry|Neighbors Serving Neighbors. https://www.midnorthfoodpantry.org/.

[58] Moguel E., García-Alonso J., Laso S. (2020). YourPantry: Food Monitoring through Pantry Analysis Using the Smartphone and Making Use Machine Learning and Deep Learning Techniques. Commun. Comput. Inf. Sci..

[59] Plentiful Food Pantry Application, Home. https://www.plentifulapp.com/.

[60] Pichardo M., Samuels G., O’Leary B., Bayiokos P., Kapiti A., Asija S., Coppola J.F. Service-learning project for computing students: Creating a mobile app for a non-profit agency. Proceedings of the 2016 IEEE Long Island Systems, Applications and Technology Conference.

[61] Rogus S., Guthrie J.F., Niculescu M., Mancino L. (2020). Online Grocery Shopping Knowledge, Attitudes, and Behaviors among SNAP Participants. J. Nutr. Educ. Behav..

[62] Schoch E., Choi A.M.L.A., Lee H., Connor S., Rose E.J. The food locker: An innovative, user-centered approach to address food insecurity on campus. Proceedings of the 37th ACM International Conference on the Design of Communication.

[63] Scott C., Biediger-Friedman L., Crixell S., Markides B. (2020). P164 Rewiring Texas WIC: Informing the Development of a Chatbot to Strengthen Enrollment in Texas. J. Nutr. Educ. Behav..

[64] Sewald C.A., Kuo E.S., Dansky H. (2018). Boulder Food Rescue: An Innovative Approach to Reducing Food Waste and Increasing Food Security. Am. J. Prev. Med..

[65] PantryWorx|Food Pantry Tracking Software. http://www.pantryworx.com/.

[66] Stotz S., Lee J.S., Hall J. (2018). A mixed-methods evaluation using low-income adult Georgians’ experience with a smartphone-based eLearning nutrition education programme. Public Health Nutr..

[67] Support The Online Food Pantry on GAgives. Mightycause. https://www.mightycause.com/organization/Theonlinefoodpantry.

[68] Traub M., Furbish S., Havens E., Ferris A.G. (2020). P88 SNAP4CT.org: An Online Nutrition Education Platform for CT SNAP Recipients. J. Nutr. Educ. Behav..

[69] Ufot J., Esterline A., Bryant K.S. Inventory Control at a University Food Pantry Using an MVC Software Pattern and Data Visualization. Proceedings of the SoutheastCon 2021.

[70] Volunteer Management Software for Food Banks|Volgistics. https://www.volgistics.com/food-banks.htm.

[71] Weinstein O., Donovan K., McCarthy A.C., Hiralall L., Allen L., Koh W., Apovian C.M. (2021). Nourishing Underserved Populations Despite Scarcer Resources: Adaptations of an Urban Safety Net Hospital During the COVID-19 Pandemic. Am. J. Public Health.

[72] Zhang Q., Zhang J., Park K., Tang C. (2021). App Usage Associated With Full Redemption of WIC Food Benefits: A Propensity Score Approach. J. Nutr. Educ. Behav..

[73] Zimmer M.C., Beaird J., Steeves E.T.A. (2020). WIC Participants’ Perspectives about Online Ordering and Technology in the WIC Program. J. Nutr. Educ. Behav..

[74] Pantri. https://pantri.com/.

[75] Mills G. (2015). Understanding the Rates, Causes, and Costs of Churning in the Supplemental Nutrition Assistance Program (SNAP).

[76] Marcolino M.S., Oliveira J.A.Q., D’Agostino M., Ribeiro A.L., Alkmim M.B.M., Novillo-Ortiz D. (2018). The Impact of mHealth Interventions: Systematic Review of Systematic Reviews. JMIR mHealth uHealth.

[77] SWAP: Overview of a Stoplight Nutrition System for Food Banks and Food Pantries. Hunger and Health. https://hungerandhealth.feedingamerica.org/resource/swap-development-stoplight-nutrition-system-food-banks-food-pantries/.

[78] Remley D., Kaiser M., Osso T. (2013). A Case Study of Promoting Nutrition and Long-Term Food Security Through Choice Pantry Development. J. Hunger. Environ. Nutr..

[79] Remley D.T., Zubieta A.C., Lambea M.C., Quinonez H.M., Taylor C. (2010). Spanish- and English-Speaking Client Perceptions of Choice Food Pantries. J. Hunger. Environ. Nutr..

[80] Nica-Avram G., Harvey J., Smith G., Smith A., Goulding J. (2020). Identifying food insecurity in food sharing networks via machine learning. J. Bus. Res..

[81] FEMA—Emergency Management Institute (EMI) Course|IS-111. A: Livestock in Disasters. https://training.fema.gov/is/courseoverview.aspx?code=is-111.a.

[82] PrepTalks. https://www.fema.gov/emergency-managers/practitioners/preptalks.

[83] Hicks S., Duran B., Wallerstein N., Avila M., Belone L., Lucero J., Magarati M., Mainer E., Martin D., Muhammad M. (2012). Evaluating Community-Based Participatory Research to Improve Community-Partnered Science and Community Health. Prog. Community Health Partnersh. Res. Educ. Action.

[84] Lucero J., Wallerstein N., Duran B., Alegria M., Greene-Moton E., Israel B., Kastelic S., Magarati M., Oetzel J., Pearson C. (2016). Development of a Mixed Methods Investigation of Process and Outcomes of Community-Based Participatory Research. J. Mix. Methods Res..

[85] Schnall R., Rojas M., Bakken S., Brown W., Carballo-Dieguez A., Carry M., Gelaude D., Mosley J.P., Travers J. (2016). A user-centered model for designing consumer mobile health (mHealth) applications (apps). J. Biomed. Inform..

[86] Burke J.G., Jones J., Yonas M., Guizzetti L., Virata M.C., Costlow M., Elizabeth M. (2013). PCOR, CER, and CBPR: Alphabet Soup or Complementary Fields of Health Research?. Clin. Transl. Sci..

[87] Luger T.M., Hamilton A.B., True G. (2020). Measuring Community-Engaged Research Contexts, Processes, and Outcomes: A Mapping Review. Milbank Q..

[88] Hauser K. (2019). Digital Tools for Food Security and Resilience: Findings and Recommendations for Burkina Faso. https://www.usaid.gov/sites/default/files/documents/1867/Burkina-Faso-Trip-Report-8.8.19.pdf.

