# Supplementary material for: Moving Food Assistance into the Digital Age: A Scoping Review
Source: Int J Environ Res Public Health. 2022 Jan 25;19(3):1328. doi: 10.3390/ijerph19031328 (PMC8835246; doi:10.3390/ijerph19031328)
Supplement: Supplementary file 1 [file ijerph-19-01328-s001.zip › ijerph-1527153-supplementary.pdf]

International Journal of
Environmental Research and Public Health

MDPI

Table S1. Search term example: SCOPUS

**SCOPUS Search Strategy**		
**Concept**	**Search Queury**	**Results**
#1: food assistance	“Food assistance” OR “Food Supply” or “food bank” or “free food” or “#freefood” “food security” or “food insecurity” or “food kitchen” or “food pantry” or “soup kitchen” or “community kitchen” or “food distribution” or “food box” or “SNAP” or “WIC” or “food aid” or “hunger” or “food first responder” or “feed the hungry” or “free meal” or “community pantr*” or "food service" OR "meal service" OR "community meal" or “foodbank” or “food stamp” or “emergency food”	15,495
#2: app features	“inventory” or “management” or “volunteer” or “training” or “project management” or “education” or “messaging” or “chat” or “preparedness” or “organization” or “command” or “communications” or “ordering” or “guidelines” or “features” or “client” or “tracking” or “dashboard” or “report” or “track” or “calendar” or “recruitment” or “choice” or “echoice” or “smart choice” or “machine learning” or “forecasting”	20,922,785
#3: Names of grey literature food pantry apps	“Pantri” or “smart choice” or “pantry worx” or “link2feed” or “food pantry manager” or “food pantry helper” or “pantry soft” or “the online food pantry” or “Flemington food pantry” or “lakeview pantry” or “plentiful” or “Connecticut food bank” or “abundance”	312
#4: digital	App OR apps OR digital OR digitally OR online OR "on-line" OR internet* OR "web based" OR website* OR virtual* OR blog* OR "chat room*" OR chatbot* OR crowdsourc* OR "crowd sourc*" OR cyber OR "electronic mail" OR email OR "e-mail*" OR "ICT" OR "information and communication technolog*" OR "information communication technolog*" OR "information technolog*"OR "instant messag*" OR "IOT" OR "e health" OR ehealth OR mHealth OR "mobile health" OR "mobile phone*" OR "cell phone*" OR cellphone* OR "cellular phone*" OR "smart phone*" OR smartphone* OR "phone screen*" OR android* OR ipad* OR iphone* OR "social media" OR "social network*" OR facebook OR Instagram OR "text	4,256,890

	messag*" OR texting OR tweet* OR twitter OR sms OR snapchat* OR Zoom* OR "google meet*" OR Webex OR "search engine*" OR "tablet system*" OR "mobile application*" OR "mobile device*" OR "mobile technolog*" OR "mobile comput*" OR "apple application*" OR "device application*" OR "phone application*" OR "computing application*" OR "Microsoft application*" OR "mac application*" OR "google application*" OR "chat application*" OR "messaging application*" OR "interactive application*" OR "tablet application*" OR "computer application*" OR "web application*" OR "food application*" OR "nutrition application*" OR "software application*" OR "software design"	
#5: Final Result	(#1 OR #2 OR #3) AND (#4) AND Date Limit: 2010 to Current	764

Table S2. Tools by implemented features.

**Tool Name**	**Client Choice**	**Client Tracking**	**Client Training**	**Communications**	**Data Analytics**	**Distribution Event Calendar**	**Emergency Preparedness**	**Donations Tracker**	**Grants Tracker**	**Inventory Management**	**Nutrition Guidelines/Education**	**Online Ordering**	**Pantry Locator**	**Prediction of Food Insecurity**	**Report Exporting**	**Social Media Sharing**	**Staff Training**	**Volunteer Management**	**Volunteer Recruitment**
Abundance Boston													x						
Bertrand et al.				x															
CHEW											x	x							
Coffiino et al.												x							
CT Food Bank Mobile Pantry Schedule						x							x						
Decision Support System										x									
Emergency Food Network				x															

Flemington Food Pantry Ordering	x											x							
Food eTalk											x						x		
Food Pantry Helper		x		x	x			x	x	x		x			x			x	
Food Pantry Manager		x			x							x			x			x	
Food Rescue Robot								x		x								x	x
Hamad et al.		x																	
Healthy Pantry Program											x								
Herron et al.				x															
Lakeview Pantry Online Market	x	x								x		x							
Link2Feed		x			x					x					x			x	
Martin et al.												x							
Maya, the Texas WIC ChatBot				x															
Midnorth Food Pantry																		x	
my WIC Family											x						x		
NEED2FEED																		x	
Nourishing Our Community											x								
Pantri		x		x	x	x				x	x			x	x			x	
Pantry Soft		x								x		x			x				
Pantry Worx		x								x					x				
Plentiful App		x											x		x				
Rogus et al.												x							
Scott et al.		x																	
Smart Choice Pantry										x		x			x				
SNAP4CT											x						x		
The Food Locker												x							

The Online Food Pantry		x						x		x		x						x	
Ufot et al.										x									
VeggieBook											x						x		
Volgistics																		x	
WICShopper												x							
YourPantry										x									
Zimmer et al.												x							
